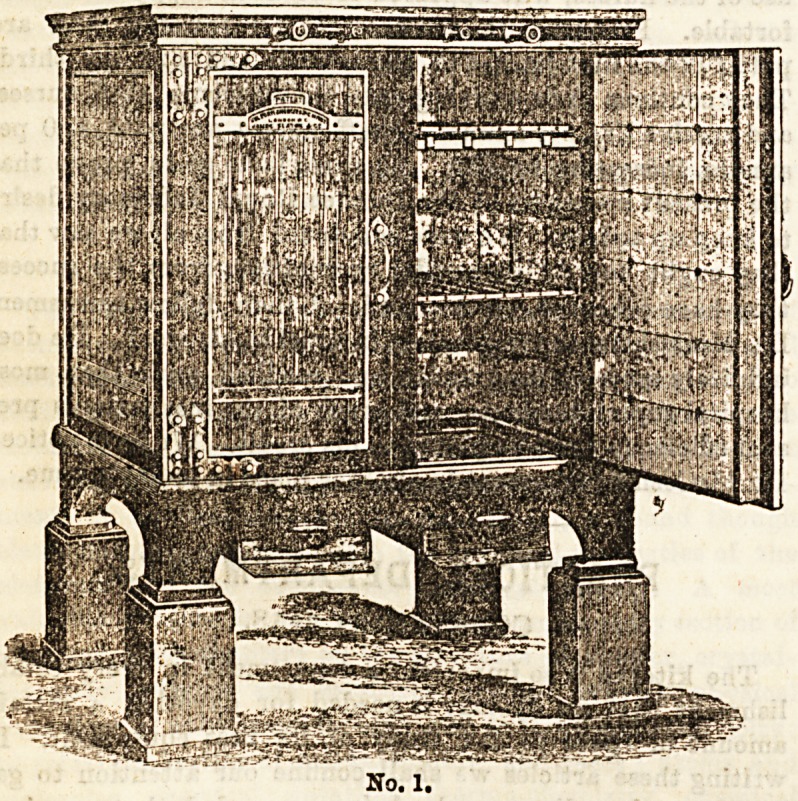# Cooking by Gas

**Published:** 1893-01-07

**Authors:** 


### PRACTICAL DEPARTMENTS.

### COOKING BY GAS.

The kitchen is so important a department in every establishment that no apology is needed for devoting a certain amount of space to the study of its best equipment. In writing these articles we shall confine our attention to gas cooking and appliances only, being persuaded that gas is a far better age ntin cooking than coal, and, indeed, so much easier is it to regulate gas to the required heat that where it is used the excuse for bad cooking is reduced to a minimum.

The dietetic superiority of well-cooked food need hardly be entered upon here, but nevertheless we must draw attention to one fundamental advantage of gas in this respect, which will be readily appreciated when pointed out. This is that by roasting in gas ovens the meat loses none of its gravy, with which it freely parts in coal cooking, either before the fire or in the oven. The reason of this arises from the unvarying heat supplied to all parts of the joint at once, which obviates the unequal contraction and expansion of the tissues inseparable from the best methods of coal cooking.

#### THE ADVANTAGES OF GAS.

The advantages from the use of gas are: (1) economy; (2) cleanliness; (3) convenience; and (4) the lower temperature of the kitchen. These are all undeniable. Economy is secured by the use of the gas only at such times when cooking is actually going on, and by the rapidity with which the requisite heat is obtained. It is thus practically more economical than coal, a fact which experience will prove. We have ourselves met with few cooks who, when free from supervision, will regulate the use of coal economically in the kitchen. Turning the gas off is so little troublesome that difficulty in this direction is seldom encountered. Far less expenditure of time is required to keep the utensils and fittings of gas cooking clean, and for this reason economy in the employment of labour can be effected in large establishments. The cleanliness of the method is obvious, and needs no comment.

On the score of convenience we cannot speak too strongly. Only those who have had actual experience in cooking can realise the advantages to the full extent, and it must be a matter of wonder to those who cook by gas habitually how anything like a steadily good result can be obtained where coal is employed. In the employment of gas there is no tedious waiting until the right degree of heat is attained; no accidental lowering of the fire at a critical moment; no checking of heat by the necessary addition of fuel, or equally inconvenient over-heating, requiring the utmost vigilance on the part of the cook. There is no smoke where the grill is employed, but a complete control over the apparatus, which gives a uniformity of result, and a possibility of really scientific accuracy. Such advantages serve to render cooking a positive pleasure, and the luxury of comparative coolness in

No. 1.

the kitchen is by no means the least of the benefits gas confers. Having thus enumerated the advantages of gas, we must not ignore objections, which are frequently raised.

### Objections to its Use.

One objection very generally entertained is the danger attendant on its use by servants, arising from the possibility of the gas being accidentally turned on when not alight. Modern improvements have entirely removed this danger. A flash light can be supplied to each apparatus, which mechanically lights the gas simultaneously with its being turned on. Of course this precaution is not universally used, but where it is not those employers are much to blame who do not have the turncocks clearly marked "off" and "on," so that the point of danger is kept constantly under notice. Smell and taste we have heard raised as objections to the method. When complaints of the sort are made we are inclined to think that they have arisen from defective apparatus. The employment of the best fittings will remove all objection, and were it not so the system would not meet with such universal approval in large hotels and restaurants, which depend on their reputation for excellence of cooking and the comfort of their guests. In the kitchen of the Hotel Victoria, to which we shall refer later on, and where gas is used with great success, there is a total absence of the typical gas odour.

It would be impossible in this article to suggest the particular apparatus suitable for all requirements, as every variety and size in ranges, ovens, &c, are now to be had; but in order to illustrate practically the general principles of these, we have selected three descriptions of fittings which are suitable for introduction into any kitchen, by an enlargement or reduction in size. The illustrations have been especially prepared for us by Messrs. James Slater and Co., of High Holborn, to whose kindness we are indebted for an inspection of the fittings in working order both at Marlborough House and the Hotel Victoria, which have been supplied by them.

No. 1 represents a most complete form of oven for roasting. The joints are placed on the shelves; the dripping is collected in drawers below the oven. A row of open burners runs along all sides of the oven, inside, at its base. These are so placed as to avoid any drippings from the joints. The whole of the inside of the oven is lined with glazed tiles. The ventilation enters at the top of the hollow door, runs down the cavity, thus purposely arranged, and enters the oven through an opening at the bottom, having become thoroughly warmed in its passage. These ovens only require heating a quarter of an hour before using, and when once the right temperature is attained a less amount of gas is required to maintain it. One simple improvement only needs to be suggested in these ovens. This is that the outer row of burners should be screened in the front to prevent the possibility of the cook's apron or the kitchen cloth coming in contact with the flames, an occurrence which not infrequently happens.

*(To be continued.)*

## Figures and Tables

**No. 1. f1:**